# Clinical characteristics and risk factors in patients with SARS-CoV-2 Omicron variant infection complicated with cardiovascular diseases

**DOI:** 10.3389/fmed.2024.1383252

**Published:** 2024-05-21

**Authors:** Xiao-hua Yu, Yu-wei Liao, Ling Rong, Bi-gui Chen, Run-jun Li, Guang-kuan Zeng, Li-li Liu, Yan-bin Cao, Jian-lian Liang, Bai-ru Lai, Yan-qing Zeng, Yu-chan Huang, Li-ye Yang

**Affiliations:** ^1^Precision Medical Lab Center, People’s Hospital of Yangjiang, Yangjiang, China; ^2^Department of Respiratory and Critical Care Medicine, Yangjiang People’s Hospital, Yangjiang, China; ^3^Department of Critical Care Medicine, People’s Hospital of Yangjiang, Yangjiang, China

**Keywords:** SARS-CoV-2, Omicron variant, clinical characteristics, China, cardiovascular diseases

## Abstract

**Objective:**

To investigate the clinical characteristics and risk factors of patients with SARS-CoV-2 Omicron variant infection complicated with cardiovascular diseases.

**Methods:**

A retrospective analysis of general clinical data was conducted on patients with SARS-CoV-2 omicron infection complicated with hypertension, coronary heart disease, and heart failure admitted to one hospital in Guangdong Province from December 1, 2022, to February 28, 2023. Clinical symptoms, laboratory tests, imaging examinations, treatment, and clinical outcomes were collected. Multivariate logistic regression analysis was used to analyze the risk factors for mortality in patients with SARS-CoV-2 Omicron variant infection complicated with cardiovascular diseases. ROC curves were drawn to evaluate the predictive value of CRP, D-dimer, and CK-MB in predicting the risk of death.

**Results:**

A total of 364 confirmed cases were included, divided into the asymptomatic group, mild to moderate group, and severe to critically ill group based on the symptoms of COVID-19. There were 216 males (59.34%) and 148 females (40.66%), with a median age of 75 years. The differences between the three groups in terms of sex and age were statistically significant (*p* < 0.05). The top three underlying diseases were hypertension (288 cases, 79.12%), coronary heart disease (100 cases, 27.47%), and diabetes (84 cases, 23.08%). The differences in unvaccinated and triple-vaccinated patients among the three groups were statistically significant (*p* < 0.05). The common respiratory symptoms were cough in 237 cases (65.11%) and sputum production in 199 cases (54.67%). In terms of laboratory tests, there were statistically significant differences in neutrophils, lymphocytes, red blood cells, C-reactive protein, D-dimer, aspartate aminotransferase, and creatinine among the three groups (*p* < 0.05). In imaging examinations, there were statistically significant differences among the three groups in terms of unilateral pulmonary inflammation, bilateral pulmonary inflammation, and bilateral pleural effusion (*p* < 0.05). There were statistically significant differences among the three groups in terms of antibiotic treatment, steroid treatment, oxygen therapy, nasal cannula oxygen inhalation therapy, non-invasive ventilation, and tracheal intubation ventilation (*p* < 0.05). Regarding clinical outcomes, there were statistically significant differences among the three groups in terms of mortality (*p* < 0.05). Multivariate logistic regression analysis showed that CRP (OR = 1.012, 95% CI = 1.004–1.019) and D-dimer (OR = 1.117, 95% CI = 1.021–1.224) were independent risk factors for patient mortality. The predictive value of CRP, D-dimer, and CK-MB for the risk of death was assessed. D-dimer had the highest sensitivity (95.8%) in predicting patient mortality risk, while CRP had the highest specificity (84.4%).

**Conclusion:**

For patients with COVID-19 and concomitant cardiovascular diseases without contraindications, early administration of COVID-19 vaccines and booster shots can effectively reduce the mortality rate of severe cases. Monitoring biomarkers such as CRP, D-dimer, and CK-MB and promptly providing appropriate care can help mitigate the risk of mortality in patients.

## Introduction

COVID-19 (corona virus disease 2019, COVID-19) is an infectious disease caused by the novel coronavirus (SARS-CoV-2). As of December 31, 2023, the cumulative number of infections worldwide has exceeded 750 million, and the number of deaths exceeded 7 million (1). At the same time, the novel coronavirus continues to undergo evolution and mutation, giving rise to variant strains such as Alpha (B.1.1.7), Beta (B.1.351), Gamma (P.1), Delta (B.1.617.2), and Omicron (B.1.1.529). WHO divides the current variants into variant strain of concern variant (variants of concern, VOC) and variant strain of interest (variant strain of interest, VOI).

The Omicron variant (B.1.1.529) was first detected in South Africa in November 2021, classified as a Variant of Concern (VOC), and named the Omicron variant (2). Subsequently, the Omicron variant (B.1.1.529) was identified in Tianjin, China, in December 2022 (3). Between December 2022 and February 2023, the Omicron variant rapidly spread across China, including Guangdong province, marking the first major COVID-19 epidemic in Guangdong. The population in Guangdong lacked natural immunity to the Omicron variant. The types of COVID-19 vaccines administered in China differed from those used in Europe and the United States. Research on cardiovascular disease combined with the Omicron variant in Guangdong Province was scarce. The Omicron variant of SARS-CoV-2 exhibited heightened transmissibility compared to previous variants but generally caused less severe illness. Nonetheless, individuals with cardiovascular diseases, such as coronary heart disease, hypertension, and heart failure, faced elevated risks due to their underlying health conditions. While data on Omicron and cardiovascular disease outcomes continue to emerge, it remains imperative to investigate the clinical characteristics and risk factors of Omicron variants in patients with cardiovascular diseases for early identification of high-risk individuals (4, 5). This study analyzed the clinical features and risk factors of patients with Omicron variant infections and cardiovascular diseases in Yangjiang city from December 1, 2022, to February 28, 2023, aiming to lay a groundwork for the early detection and diagnosis of at-risk patients.

## Materials and methods

### Clinical data

A total of 364 patients with comorbid hypertension, coronary heart disease, and heart failure, who were admitted to People’s Hospital of Yangjiang in Guangdong Province, China, from December 1, 2022, to February 28, 2023, were included as study subjects. Of these patients, 216 were male and 148 were female. SARS-CoV-2 nucleic acid testing was used for diagnosis, and a positive result confirmed the cases. General clinical data of the patients were collected, including age, sex, medical history, clinical symptoms, laboratory test results, treatment, and prognosis. This study was approved by the Ethics Review Committee of People’s Hospital of Yangjiang (No. 20230003).

### Diagnostic and classification criteria

All enrolled patients met the diagnostic criteria for COVID-19 outlined in the “Diagnosis and Treatment Protocol for Novel Coronavirus Pneumonia (Trial Version 5)” issued by the National Health Commission of China (6). Patients meeting the following criteria were included in the study: (1) all enrolled patients tested positive for SARS-CoV-2 nucleic acid, (2) aged 18 years or older, (3) with a medical history or a confirmed diagnosis upon admission of conditions such as hypertension, coronary heart disease, heart failure, and arrhythmia. Patients with incomplete clinical data, including age, gender, vaccination status, and comorbidities, were excluded from the study. Clinical classification upon admission was as follows: (1) mild: primarily presenting with upper respiratory tract infection symptoms such as dry throat, sore throat, cough, fever, etc.; (2) moderate: persistent high fever (>3 days) and/or cough, dyspnea, with a respiratory rate (RR) <30 breaths per minute, resting oxygen saturation > 93%, and characteristic imaging findings of COVID-19 pneumonia; (3) severe: meeting any of the following criteria in adults and unable to be explained by causes other than COVID-19 infection: (a) presence of dyspnea with a respiratory rate ≥30 breaths per minute; (b) resting oxygen saturation ≤93%; (c) arterial partial pressure of oxygen (PaO_2_) to fractional inspired oxygen concentration (FiO_2_) ratio ≤300 mmHg (1 mmHg = 0.133 kPa); (d) progressive worsening of clinical symptoms, with lung imaging showing >50% lesion progression within 24–48 h; (4) critical: meeting any of the following conditions: (a) respiratory failure requiring mechanical ventilation; (b) shock; (c) organ failure requiring ICU monitoring and treatment. Among them, some patients tested positive for SARS-CoV-2 nucleic acid but had no respiratory system clinical symptoms were classified as asymptomatic infections. Based on the clinical classification, asymptomatic infections were grouped separately, while mild and moderate cases were grouped together, and severe and critical cases were grouped together.

### Laboratory tests

Laboratory test results of the patients were collected at admission, including white blood cell count (WBC), lymphocyte count (LYM), neutrophil count (NEUT), red blood cell count (RBC), hemoglobin level (HB), C-reactive protein (CRP), D-dimer, cardiac injury markers [including creatine kinase-MB (CK-MB)], liver function, and kidney function.

### Statistical methods

Statistical description and analysis of the research data were conducted using SPSS 26.0 software. Non-normally distributed continuous data were presented as median with interquartile range (Q1, Q3), and between-group comparisons were performed using rank-sum tests. Categorical data were presented as percentages (%), and between-group comparisons were conducted using Pearson’s chi-square test or Fisher’s exact test, depending on the data characteristics. Logistic regression was used to explore the risk factors associated with mortality, the diagnostic value of risk factors in predicting patient mortality was analyzed using ROC curves. A *p*-value <0.05 was considered statistically significant for differences.

## Results

### Clinical data and clinical symptoms

A total of 364 cases of COVID-19 patients were included, with 216 males (59.34%) and 148 females (40.66%). The median age was 75 years (range: 65–83 years). Among the COVID-19 patients, there were 43 cases of asymptomatic infections, 289 cases of mild and moderate cases, and 32 cases of severe and critical cases. The differences between the three groups in terms of sex and age were statistically significant (*p* < 0.05). Based on the medical records of COVID-19 patients upon admission, the clinical symptoms were analyzed. The top three symptoms were cough in 237 cases (65.11%), sputum production in 199 cases (54.67%), and fever in 161 cases (44.23%). There were statistically significant differences among the three groups in terms of clinical symptoms, including fever, cough, sputum production, sore throat, poor appetite, general fatigue, dizziness, headache, and chest tightness (*p* < 0.05). The top three underlying diseases were hypertension with 288 cases (79.12%), coronary heart disease with 100 cases (27.47%), and diabetes with 84 cases (23.08%). There were statistically significant differences among the three groups in terms of the presence of heart failure and stage 5 chronic kidney diseases (*p* < 0.05). In terms of vaccination status, 118 cases (32.42%) were unvaccinated, 52 cases (14.29%) received one dose, 70 cases (19.23%) received two doses, and 124 cases (34.07%) received three doses. The differences among the three groups in terms of unvaccinated and triple-vaccinated patients were statistically significant (*p* < 0.05) (Table 1).

**Table 1:** General clinical data and signs of the patients in the three groups.

	Total (*n* = 364)	Asymptomatic group (*n* = 43)	Mild and medium (*n* = 289)	Severe and critical (*n* = 32)	*H*/*X*^2^	*p*-value
**Gender [*n*, (%)]**						
Male	216 (59.34)	28 (65.11)	163 (56.40)	25 (78.13)	6.309	0.041^b^
Female	148 (40.66)	15 (34.89)	126 (43.60)	7 (21.87)		
Age (year)	75 (65，83)	70 (64，78)	76 (65，83)	82 (69，86)	8.359	0.015^ac^
**Symptoms and signs**						
Fever, °C	161 (44.23)	0	138 (47.75)^b^	23 (71.88)	45.469	<0.001^abc^
37.3–38.0	57 (15.66)	0	52 (17.99)	5 (15.63)	9.150	0.010^a^
38.1–39.0	75 (20.60)	0	64 (22.15)	11 (34.38)	15.246	<0.001^ac^
>39.0	29 (7.97)	0	22 (7.61)	7 (21.86)	12.180	0.002^c^
Cough	237 (65.11)	0	207 (71.63)	30 (93.75)	96.934	<0.001^abc^
Expectoration	199 (54.67)	0	173 (59.86)	26 (81.25)	64.126	<0.001^ac^
Runny nose	7 (1.92)	0	5 (1.73)	2 (6.25)	3.230	0.156
Stuffy nose	5 (1.37)	0	4 (1.38)	1 (3.13)	1.407	0.450
Sore throat	46 (12.64)	0	43 (14.88)	3 (9.38)	7.844	0.020^a^
Decreased food appetite	86 (23.63)	0	78 (26.99)	8 (25.00)	15.147	0.001^c^
Diarrhea	7 (1.92)	0	6 (2.08)	1 (3.13)	0.997	0.596
Lacking in strength	29 (7.97)	0	28 (9.69)	1 (3.13)	6.014	0.041
Dizziness headache	103 (28.30)	0	102 (35.29)	1 (3.13)	33.937	<0.001^ab^
Myalgia	10 (2.75)	0	9 (3.11)	1 (3.13)	0.986	0.700
Abdominal pain	2 (0.55)	0	2 (0.69)	0	0.671	1.000
Nausea	23 (6.32)	0	22 (7.61)	1 (3.13)	4.271	0.091
Vomiting	34 (9.34)	0	32 (11.07)	2 (6.25)	6.545	0.034
Chest tightness	92 (25.28)	0	86 (29.76)	6 (18.75)	18.341	<0.001^ac^
**With the underlying disease [*n*, (%)]**						
Tumor	25 (6.97)	3 (6.98)	20 (6.92)	2 (6.25)	0.096	1.000
**Angiocardiopathy**						
Hypertension	288 (79.12)	35 (81.40)	228 (78.90)	25 (78.13)	0.163	0.922
Coronary disease	100 (27.47)	8 (18.60)	85 (29.41)	7 (21.88)	2.726	0.253
Heart-failure	70 (19.23)	0	59 (20.41)	11 (34.38)	15.224	<0.001^ac^
Arhythmia	27 (7.42)	5 (11.63)	20 (6.92)	2 (6.25)	1.431	0.529
**Respiratory disease**						
COPD	21 (5.77)	1 (2.32)	16 (5.54)	4 (12.50)	3.257	0.179
Bronchial asthma	2 (0.55)	0	1 (0.35)	1 (3.13)	3.680	0.182
**Kidney disease**						
Chronic kidney disease, stage 3	1 (0.27)	0	1 (0.35)	0	1.589	1.000
Chronic kidney disease, stage 4	2 (0.55)	0	2 (0.70)	0	0.671	1.000
Chronic kidney disease, stage 5	14 (3.85)	3 (6.98)	7 (2.42)	4 (12.50)	8.199	0.011^b^
**Hepatic disease**						
Cirrhosis	3 (0.82)	0	3 (1.04)	0	0.330	1.000
Chronic hepatitis	7 (1.92)	1 (2.32)	6 (2.08)	0	0.407	1.000
**Metabolic disease**						
Diabetes mellitus	84 (23.08)	15 (34.88)	69 (23.88)	9 (28.13)	2.507	0.286
Thyroid disease	10 (2.75)	2 (4.65)	8 (2.77)	0	1.188	0.590
**Vaccine status [*n*, (%)]**						
Unvaccinated	118 (32.42)	4 (9.30)	101 (34.95)	13 (40.63)	12.316	0.002^ac^
**Vaccination**						
One dose	52 (14.29)	4 (9.30)	45 (15.57)	3 (9.38)	1.892	0.388
Two doses	70 (19.23)	8 (18.60)	58 (20.69)	4 (12.50)	1.075	0.584
Three doses	124 (34.07)	27 (62.79)	85 (29.41)	12 (37.50)	18.751	<0.001^a^

^a^Pairwise comparisons showed significant differences between the asymptomatic group versus the mild and medium groups, *p* < 0.05. ^b^Pairwise comparisons showed significant differences between the mild and medium groups versus severe and critical groups, *p* < 0.05. ^c^Pairwise comparisons showed significant differences between the asymptomatic group versus the severe and critical groups, *p* < 0.05. Categorical data are presented as number of patients (*n*) and percentage (%). Differences between categorical data were determined using the Pearson chi-square test or the Fisher’s exact test. If significant, pairwise analysis was performed using the same test. Measurement data with non-normal distribution are expressed in M (Q1, Q3), Groups were compared using the Kruskal Wallis rank-sum test.

### Laboratory examinations

The first laboratory test results upon admission of COVID-19 patients were collected, and statistical analysis was performed on the laboratory test results among the three groups. There were statistically significant differences among the three groups in terms of neutrophils, lymphocytes, red blood cells, C-reactive protein, D-dimer, aspartate aminotransferase, and creatinine levels (*p* < 0.05) (Table 2).

**Table 2:** Laboratory findings of the three groups.

	Asymptomatic (*n* = 43)	Mild and medium (*n* = 285)	Severe and critical (*n* = 32)	*H*	*p*-value
WBC, ×10^9^/L, (M, IQR)	6.05 (5.17, 6.88)	5.66 (4.49, 7.98)	7.50 (4.55, 11.34)	5.419	0.067
NEUT, ×10^9^/L, (M, IQR)	3.97 (3.13, 4.93)	4.03 (2.83, 6.24)	6.53 (3.62, 9.31)	9.943	0.007^bc^
LYM, ×10^9^/L, (M, IQR)	1.19 (0.94, 1.75)	0.90 (0.58, 1.32)	0.66 (0.40, 1.12)	20.171	<0.001^ac^
RBC, ×10^9^/L, (M, IQR)	4.42 (3.84, 4.83)	4.05 (3.36, 4.58)	4.19 (3.32, 4.65)	6.566	0.038^a^
Hb (g/L), (M, IQR)	124.00 (112.00, 137.00)	119.00 (99.50, 133.00)	122.5 (96.50, 139.50)	3.27	0.195
CRP (mg/L), (M, IQR)	7.32 (3.12, 17.79)	14.44 (4.98, 48.09)	47.15 (13.23, 156.56)	28.342	<0.001^abc^
D-dimer (μg/mL), (M, IQR)	0.47 (0.23, 0.95)	0.48 (0.26, 0.87)	1.01 (0.49, 3.92)	14.682	0.001^bc^
CK-MB (U/L), (M, IQR)	16.00 (12.50, 20.00)	15.90 (12.60, 21.00)	18.95 (14.60, 30.38)	4.714	0.095
AST (μ/L), (M, IQR)	24.60 (19.60, 30.60)	31.50 (23.15, 46.35)	41.45 (23.53, 57.18)	14.771	0.001^ac^
ALT (μ/L), (M, IQR)	20.30 (17.20, 28.10)	24.80 (16.95, 36.55)	29.35 (18.25, 40.00)	3.705	0.157
ALP (μ/L), (M, IQR)	76.00 (60.00, 92.00)	70.00 (59.00, 88.50)	74.00 (62.25, 83.00)	1.674	0.433
Cr (μmol/L) (M, IQR)	79.00 (64.00, 90.00)	83.00 (62.00, 116.00)	95.00 (73.75, 242.75)	6.052	0.049^c^

^a^Pairwise comparisons showed significant differences between the asymptomatic group versus the mild-medium groups, *p* < 0.05. ^b^Pairwise comparisons showed significant differences between the mild-medium group versus severe-critical group, *p* < 0.05. ^c^Pairwise comparisons showed significant differences between the asymptomatic group versus the severe-critical group, *p* < 0.05. Measurement data with non-normal distribution are expressed in M (Q1, Q3), Groups were compared using the Kruskal Wallis rank-sum test.

### Radiological examinations

The first radiological examination results upon admission of COVID-19 patients were collected, but some patients did not have radiological results due to either mild or severe conditions. Statistical analysis was performed on patients who had completed chest CT scans. There were statistically significant differences among the three groups in terms of unilateral pulmonary inflammation, bilateral pulmonary inflammation, and bilateral pleural effusion (*p* < 0.05) (Table 3).

**Table 3:** Characteristics of the first chest CT examination in the three groups.

	Total (*n* = 247)	Asymptomatic (*n* = 1)	Mild and medium (*n* = 217)	Severe and critical (*n* = 29)	*X* ^2^	*p*-value
**Results of the first chest CT examination [*n*/(%)]**						
No obvious abnormality	12 (4.86)	0	12 (5.53)	0	2.480	1.000
Unilateral lung inflammation	23 (9.31)	0	20 (9.22)	3 (10.35)	14.001	0.001^b^
Bilateral lung inflammation	166 (67.21)	1 (100.00)	139 (64.06)	26 (89.66)	18.521	<0.001^b^
Unilateral pleural effusion	19 (7.69)	0	18 (8.30)	1 (3.45)	6.466	0.166
Bilateral pleural effusion	11 (4.45)	0	9 (4.15)	2 (6.90)	13.988	0.002^b^

^b^Pairwise comparisons showed significant differences between the mild-medium groups versus severe-critical groups, *p* < 0.05. Categorical data are presented as number of patients (*n*) and percentage (%). Differences between categorical data were determined using the Pearson chi-square test or the Fisher’s exact test. If significant, pairwise analysis was performed using the same test.

### Treatment status

The treatment status of COVID-19 patients was collected and subjected to statistical analysis. There were statistically significant differences among the three groups in terms of antibiotic treatment, steroid treatment, oxygen therapy, nasal cannula inhalation, non-invasive ventilation, and tracheal intubation ventilation (*p* < 0.05) (Table 4).

**Table 4:** Treatment of the patients in the three subgroups.

Treatment plan	Total (*n* = 364)	Asymptomatic (*n* = 43)	Mild and medium (*n* = 289)	Severe and critical (*n* = 32)	*X* ^2^	*p*-value
Antibiotic [*n*/(%)]	161 (44.23)	1	134 (46.38)	26 (81.25)	48.924	<0.001^abc^
Antiviral [*n*/(%)]	35 (9.62)	1	29 (10.04)	5 (15.63)	4.185	0.123
Prednisone [*n*/(%)]	66 (18.13)	0	52 (17.99)	14 (43.75)	23.675	<0.001^abc^
No oxygen [*n*/(%)]	148 (40.66)	43 (100.00)	105 (36.33)	0	86.925	<0.001^abc^
Nasal catheter inhalation [*n*/(%)]	199 (54.67)	0	180 (62.28)	19 (59.38)	58.906	<0.001^ac^
Non-invasive assisted ventilation [*n*/(%)]	9 (2.47)	0	1 (0.35)	8 (25.00)	33.454	<0.001^bc^
Trachea intubation ventilation [*n*/(%)]	8 (2.20)	0	3 (1.04)	5 (15.63)	19.295	<0.001^bc^

^a^Pairwise comparisons showed significant differences between the asymptomatic group versus the mild-medium group, *p* < 0.05. ^b^Pairwise comparisons showed significant differences between the mild and medium groups versus severe-critical group, *p* < 0.05. ^c^Pairwise comparisons showed significant differences between the asymptomatic group versus the severe-critical group, *p* < 0.05. Categorical data are presented as number of patients (*n*) and percentage (%). Differences between categorical data were determined using the Pearson chi-square test or the Fisher’s exact test. Pairwise analysis was performed using the same test if the difference was significant.

### Clinical outcomes

Statistical analysis was conducted on the clinical outcomes of COVID-19 patients in the three groups. Out of the three groups, 340 patients (93.41%) were discharged, while 24 patients (6.59%) died. Among the deaths, 19 cases (5.22%) were directly attributed to respiratory failure caused by COVID-19. There were statistically significant differences among the three groups in terms of mortality (*p* < 0.05) (Table 5).

**Table 5:** Clinical outcomes of the three patient groups.

	Total (*n* = 364)	Asymptomatic (*n* = 43)	Mild and medium (*n* = 289)	Severe and critical (*n* = 32)	*X* ^2^	*p*-value
**Prognosis**						
Survival [*n*/(%)]	340 (93.41)	43 (100.00)	280 (96.87)	17 (53.13)	0.983	0.554
Death [*n*/(%)]	24 (6.59)	0	9 (3.11)	15 (46.88)	50.399	<0.001^bc^
Respiratory failure	19 (5.22)	0	7 (2.42)	12 (37.50)	39.131	<0.001^bc^
Heart failure	3 (0.82)	0	2 (0.69)	1 (3.13)	2.521	0.276
Kidney failure	1 (0.28)	0	0	1 (3.13)	5.991	0.088
Multiple organ dysfunction syndrome	1 (0.28)	0	0	1 (3.13)	5.991	0.088

^b^Pairwise comparisons showed significant differences between the mild- medium groups versus severe-critical groups, *p* < 0.05. ^c^Pairwise comparisons showed significant differences between the asymptomatic group versus the severe-critical groups, *p* < 0.05. Categorical data are presented as number of patients (*n*) and percentage (%). Differences between categorical data were determined using the Pearson chi-square test or the Fisher’s exact test. Pairwise analysis was performed using the same test if the difference was significant.

### Risk factors analysis for mortality

Based on the results of univariate analysis, 12 indicators were selected for multivariate logistic regression analysis. The results showed that CRP (OR = 1.012, 95% CI = 1.004–1.019) and D-dimer (OR = 1.117, 95% CI = 1.021–1.224) were significantly associated with patient mortality (*p* < 0.05) (Table 6).

**Table 6:** Multivariate logistic regression analysis.

Parameters	*p*-value	OR (95% CI)
WBC	0.743	0.797 (0.205–3.101)
Neu	0.549	1.544 (0.373–6.395)
LY	0.414	0.365 (0.032–4.106)
CRP	0.003	1.012 (1.004–1.019)
D-dimer	0.016	1.117 (1.021–1.224)
CKMB	0.179	1.013 (0.994–1.031)
AST	0.921	1.001 (0.990–1.011)
ALT	0.966	1 (0.986–1.013)
ALP	0.612	0.995 (0.977–1.014)
CR	0.38	1.001 (0.999–1.003)
Age	0.272	1.032 (0.975–1.092)
Sex	0.374	1.833 (0.482–6.965)

### ROC diagnostic model for risk factors of mortality

**Figure 1:**
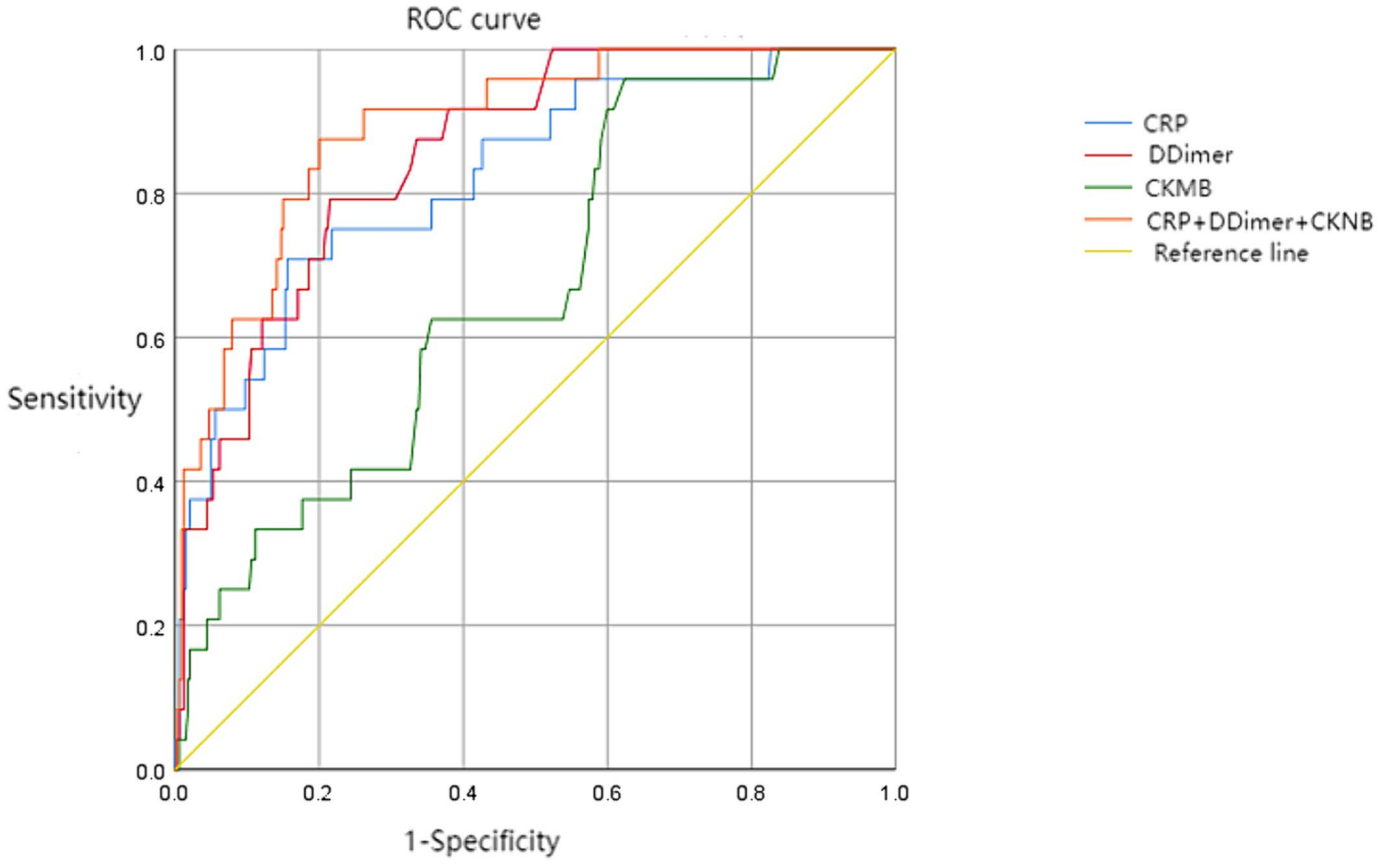
Diagnostic value of CRP, D-dimer, and CKMB for prognostic risk.

To further investigate the predictive value of CRP, D-dimer, and CKMB in assessing the risk of mortality, a ROC prediction analysis model was established using the death group as the positive sample (*n* = 24) and the survival group as the negative sample (*n* = 340). The ROC curves for each indicator were fitted using software to obtain the maximum Youden index, and the corresponding theoretical thresholds and parameters were calculated. The results showed that D-dimer had the highest sensitivity in predicting patient mortality risk, while CRP had the highest specificity in predicting patient mortality risk (Table 7 and Figure 1).

**Table 7:** ROC curve analysis of the predictive value of different indicators.

Variables	AUC	Sensitivity	Specificity	Youden index	Cut point
CRP	0.823	0.708	0.844	0.552	61.925
D-dimer	0.855	0.958	0.444	0.402	10.65
CKMB	0.668	0.792	0.785	0.577	0.965
CRP + D-dimer + CK-MB	0.891	0.875	0.8	0.675	—

## Discussion

During the early stage of the epidemic, COVID-19 patients progressed rapidly, and some patients experienced various complications and even multiple organ dysfunction, even leading to death (7). Epidemiological data have shown that in South Africa, infections with the Omicron variant reach 90% of all newly diagnosed COVID-19 cases within approximately 25 days (8). Studies have demonstrated that the Omicron variant has over 60 substitutions, deletions, and insertions, with some mutations being associated with increased transmissibility, disease severity, and immune escape (9). Previous research has indicated that the proportion of critically ill patients and the mortality rate are elevated in COVID-19 patients with comorbid cardiovascular diseases, which is a relevant risk factor for poor prognosis (10).

Reports by Wang et al. (11) suggest that the male-to-female ratio of confirmed COVID-19 cases is 1.06:1, indicating that both males and females are susceptible to the novel coronavirus infection. However, the incidence rate in males is slightly higher, and being male is considered a risk factor for severe disease. In this study, the percentage of males in the severe and critical groups was 78.13%, which is consistent with the above-mentioned report. Previous studies have indicated that the median age of COVID-19 cases is around 47 years (12). In this study, the median age was 75 years, which can be attributed to the inclusion criteria of this study, as all patients had comorbid cardiovascular diseases. Regarding comorbidities, the top three in this study were hypertension (79.12%), coronary heart disease (27.47%), and diabetes (23.08%); moreover, all of these patients were over 60 years old. These individuals with weakened immune function and underlying chronic diseases are more susceptible to COVID-19 infection, especially among the elderly population with comorbidities.

COVID-19 vaccination is currently an effective method for preventing COVID-19 and can generate neutralizing antibodies in the body (13). Both Pfizer and BioNTech claim that booster doses of the vaccine can provide high levels of protection against the Omicron variant. Preliminary trials have shown that compared to the two-dose vaccine, the booster dose can increase antibody protection by 25-fold, and the antiviral capacity after receiving the booster dose is equivalent to the 95% protection provided by the two-dose vaccine against the original virus strain (14). This study shows statistically significant differences (*p* < 0.05) in the three groups of patients regarding vaccination status, with the non-vaccinated proportion being as high as 40.63% in the severe and critical group compared to only 9.30% in the asymptomatic group. Severe patients also had a higher proportion of receiving treatments such as hormones, antibiotics, antivirals, and mechanical ventilation. The fact remains that vaccination proves effective in preventing disease progression and improving prognosis in patients infected with the COVID-19 virus.

Dr. Angelique Kotze (14) from the South Africa believes that infections with the Omicron variant present with mild symptoms, primarily including fatigue, muscle aches, headaches, and dry cough, but no patients have reported obvious symptoms such as loss of smell or taste, or difficulty breathing. In this study, patients mainly presented with cough, sputum production, and fever, and some patients initially had atypical symptoms, with 11.81% even being asymptomatic. Additionally, 4.86% of the cases had no apparent abnormalities on chest CT, indicating that in the early stages of COVID-19, the diversity of clinical symptoms and radiographic features can make the diagnosis of the disease more complex. In terms of laboratory examinations, compared to the asymptomatic and mild–moderate groups, the severe and critical group showed increased neutrophils, C-reactive protein, and D-dimer levels, as well as a decrease in lymphocytes, which is consistent with the literature (15). This indicates that the inflammatory response and the extent of viral infection are more severe in severe and critical patients compared to the asymptomatic, and mild–moderate groups. It also suggests that the COVID-19 virus attacks lymphocytes, leading to a decrease in their numbers, resulting in compromised immune function and secondary infections, making severe patients more prone to severe pneumonia and even multiple organ dysfunction.

In the early stages of the Omicron variant spread, epidemiological research has shown that the risk of hospitalization, severe illness, and death caused by the Omicron variant is lower than previous variants. A retrospective cohort study conducted in the United States found that compared to the Delta variant, the overall risk of hospitalization among Omicron variant cases decreased by 41% (15). Specifically, the risk of admission to the intensive care unit decreased by 50%, the risk of mechanical ventilation decreased by 64%, and the risk of death decreased by 79% (16). This study shows a mortality rate of 6.59% among patients, indicating that comorbidities are a high-risk factor for COVID-19 patient mortality, with 5.22% of deaths attributed to respiratory failure. COVID-19 infection is caused by the binding of the spike protein on the virus surface to the angiotensin-converting enzyme 2 (ACE2) receptor expressed on human airway epithelial cells (17). As ACE2 is highly expressed in alveolar cells, some researchers believe this may explain the severe alveolar damage observed after COVID-19 infection (18), which can progress to acute respiratory distress syndrome (ARDS). Other deaths attributed to heart failure and kidney failure are considered to be due to pre-existing comorbidities. Previous studies have demonstrated that C-reactive protein (CRP) has high clinical value in reflecting the severity of COVID-19 (19). In the analysis of risk factors for mortality in this study, higher levels of CRP and D-dimer were associated with increased risk of death. Elevated CRP levels indicate an excessive inflammatory response following viral infection and often suggest a poor prognosis. The significant elevation of D-dimer in severe and critical patients indicates enhanced coagulation activity and warrants early intervention measures to prevent disseminated intravascular coagulation (DIC). CK-MB plays an important clinical role in the diagnosis and risk stratification of acute myocardial infarction. Some studies have indicated that CK-MB levels are elevated beyond the normal range in severe and critical patients, and the severity of cardiac injury worsens with the clinical classification of COVID-19 (20). Therefore, early monitoring of abnormal elevation in cardiac enzyme levels is of significant importance. ROC curve analysis shown that the area under the curve for CRP, D-dimer, and CK-MB falls within the range of 0.6 to 0.9, indicating good clinical diagnostic value for prognostic risk. Moreover, increased attention should be given to the elevated risk of mortality in COVID-19 patients when the following criteria are met: CRP >61.925 mg/L, D-dimer >10.65 ug/mL, and CK-MB >0.965 U/L.

This study has several limitations. Firstly, certain cases did not undergo imaging examinations or had incomplete laboratory testing, and none of the cases were tested for cytokine levels. Secondly, the sample size of this study is small, and the patients are geographically limited to a single unit in a municipal hospital. It would be beneficial to include cases from diverse regions to augment the sample size and strengthen the article’s credibility. Furthermore, we did not perform follow-up assessments; thus, attention should be given to the long-term prognosis of these patients. In summary, patients with underlying cardiovascular diseases tend to be older, with respiratory system infections being the main clinical manifestation. Most patients have a good prognosis, while the mortality rate is higher among severe and critical patients who have not received the COVID-19 vaccine. It is advocated that patients without contraindications to vaccination should receive the COVID-19 vaccine and booster shots as early as possible to effectively enhance vaccine protection and increase the vaccination rate of booster shots, thereby reducing the mortality rate among severe patients. Paying attention to the indicators of CRP, D-dimer, and CK-MB in patients and providing relevant symptomatic treatment as early as possible can improve patient prognosis and reduce the risk of death among COVID-19 patients with underlying cardiovascular diseases.

## Data availability statement

The raw data supporting the conclusions of this article will be made available by the authors, without undue reservation.

## Ethics statement

The studies involving humans were approved by the Ethics Review Committee of People’s Hospital of Yangjiang. The studies were conducted in accordance with the local legislation and institutional requirements. The ethics committee/institutional review board waived the requirement of written informed consent for participation from the participants or the participants’ legal guardians/next of kin because This study was a retrospective study.

## Author contributions

X-hY: Data curation, Investigation, Methodology, Software, Validation, Writing – original draft, Writing – review & editing. Y-wL: Software, Writing – review & editing. LR: Investigation, Writing – review & editing. B-gC: Investigation, Writing – review & editing. R-jL: Resources, Writing – review & editing. G-kZ: Methodology, Writing – review & editing. L-lL: Resources, Writing – review & editing. J-lL: Writing – original draft. B-rL: Writing – review & editing. Y-qZ: Resources, Writing – review & editing. Y-cH: Writing – review & editing. L-yY: Conceptualization, Data curation, Funding acquisition, Methodology, Supervision, Validation, Writing – original draft, Writing – review & editing. Y-bC: ____.
